# Effects of the Aging Period and Method on the Physicochemical, Microbiological and Rheological Characteristics of Two Cuts of Charolais Beef

**DOI:** 10.3390/foods12030531

**Published:** 2023-01-25

**Authors:** Marika Di Paolo, Rosa Luisa Ambrosio, Claudia Lambiase, Valeria Vuoso, Angela Salzano, Giovanna Bifulco, Carmela Maria Assunta Barone, Raffaele Marrone

**Affiliations:** 1 Department of Veterinary Medicine and Animal Production, University of Naples Federico II, 80137 Naples, Italy; 2 Department of Agricultural Sciences, University of Naples Federico II, 80055 Portici (Na), Italy

**Keywords:** wet-aging, dry-aging, meat quality, cooking loss, fatty acids profile, food safety

## Abstract

Wet-aging (WA) and dry-aging (DA) methods are usually used in the beef industry to satisfy the consumers’ tastes; however, these methods are not suitable for all anatomical cuts. In this study, WA and DA were applied to improve the quality of two cuts of Charolais beef (*Longissimus dorsi* and *Semitendinosus*). For 60 days (i.e., 2 days, 15 days, 30 days and 60 days of sampling), a physicochemical, rheological, and microbiological analysis were performed at WA (vacuum packed; temperature of 4 ± 1 °C) and at DA (air velocity of 0.5 m/s; temperature of 1 ± 1 °C; relative humidity of 78 ± 10%) conditions. The results showed that the aging method influenced the aging loss (higher in the DA), cooking loss (higher in the WA), malondialdehyde concentration (higher in the DA) and fatty acid profile (few changes). No differences in the drip loss and color were observed, which decreased after 30 days of aging. The WBSF and TPA test values changed with increasing an aging time showing that the aging improved the tenderness of meat regardless of the aging method. Moreover, the aging method does not influence the microbiological profile. In conclusion, both WA and DA enhanced the quality of the different beef cuts, suggesting that an optimal method-time and aging combination could be pursued to reach the consumers’ preferences.

## 1. Introduction

Meat is the main source of high biological value proteins for human consumption and micro-nutrients such as iron, zinc and B vitamins [1]. In addition, beef is also a source of several bioactive compounds such as conjugated linoleic acid (CLA), taurine, creatine, betaine and carnitine [2]. The qualitative characteristics of meat such as the tenderness, flavor and juiciness are important factors to determine the acceptability and palatability of meat and they guide consumer choices [3]. In Italy, most of the animals raised for meat production are imported from France; the main breeds are Charolais and Limousine [4]. In order to satisfy consumers’ preferences, the beef industry retails the meat cuts on the middle side of the carcass such as the loin (*Longissimus dorsi*) [5]. This preference for the middle cuts has resulted in a lower utilization of the end cuts, such as the round (*Semitendinosus*) and it has intensified the retail price differences between the preferred and not-preferred meat cuts [6]. Lepper-Blilie et al. [7] and Kim et al. [8] reported that different cuts have the potential added value to replace the “preferred cuts” at a lower cost. In this regard, aging technology has become essential to enhance meat tenderness and flavor and to answer to the high demand and the quality expectation of consumers [9,10]. Meat aging is a complex process that includes proteolysis, lipolysis, moisture loss and interaction with microorganisms, which begins immediately after animal slaughter [10,11]. As reported by Xu et al. [12], nowadays, wet-aging (WA) and dry-aging (DA) are the two main forms of aging methods used in the beef industry (that includes local processors, food retailers and packers). The different conditions between WA and DA can result in different final qualities. In the WA method, the fresh meat is stored in a vacuum packaging bag. Moreover, WA meat loses weight, is juicier and more tender, has little or no cooler shrinkage and is preferred from an economic point of view, due to its quicker entry to the market [12]. In contrast, in the DA method, the evaporation of moisture dries the meat surface, making it inedible (forming a “crust”) and increasing the weight loss, hence resulting in a higher economic loss [8,12]. The DA process requires controlled environmental conditions regarding the drying temperature, relative humidity and airflow. Each of these parameters influences the flavor development and shelf-life of the meat, thus determining its qualities and commercial properties [13]. Moreover, during the long-time aging process, many microbes colonize the meat’s surface and the composition of the microbial community changes continuously [14]. Microbes metabolize nutrients in the meat and produce different metabolites that affect the flavor, tenderness, and rancidity of aged meat [15]. For this reason, the continuous control of aging conditions is an important strategy to reduce the risk of meat spoilage, especially for extended aging periods.

The WA and DA methods are not suitable for all anatomical cuts. Marrone et al. [16] reported that the *Semitendinosus* muscle of buffalo were not analyzable after 30 d of dry-aging because it was too dark and dry to be measured. The DA method typically requires meat marbled enough to ensure a consistent flavor and juiciness [17]. Based on our previous research [16], the purpose of this study was to use two different aging methods (i.e., WA and DA) on two different anatomical Charolais cuts (i.e., *Longissimus dorsi* and *Semitendinosus*) and to observe changes in the quality traits and microbial characteristics of the meat aged for 60 days. 

## 2. Materials and Methods 

### 2.1. Samples Preparation and Aging Methods

The study used *Semitendinosus* and *Longissimus dorsi* muscles from three bovine Charolais aged 780 ± 79.3 days and with an average live weight of 603.27 ± 60.27 kg. The animals were reared at a private farm located in southern Italy (“Le Masserie Piano Società Agricola S.r.l”, Avellino, Italy) and were slaughtered in a commercial EU-licensed slaughterhouse by exsanguination after stunning by captive bolt. The dressing percentage (i.e., the ratio of the dressed carcass weight to the weight of the live animal) was calculated. The pH of both sides of the carcasses was measured manually immediately after being slaughtered, using a portable pH meter (HI9025, Hanna Co., Villafranca Padovana, Italy) equipped with a meat puncture electrode which was inserted into a small incision (4 cm depth) in the hindquarter and forequarter (neck muscles) of the carcass. The carcasses were chilled at 4 ± 1 °C and were graded according to the European beef grading system (Regulation 103/2006, CE 1234/2007 and CE 1249/2008), using the SEUROP classification scale for conformation and a five-point scale for the fatness score.

**Figure 1:**
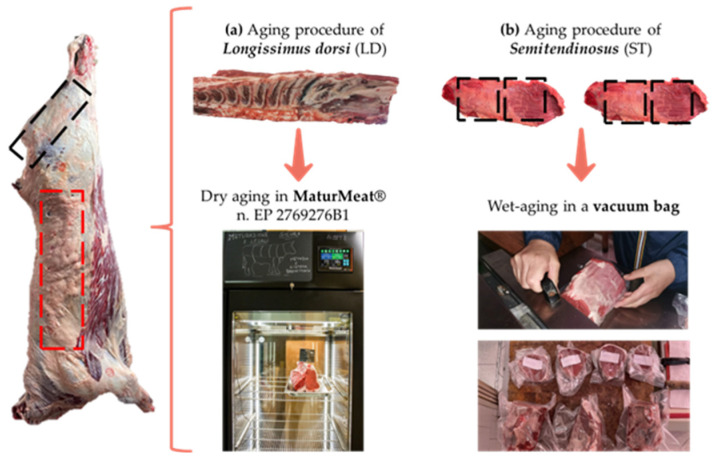
The schematic figures illustrate the dry-aging (**a**) and wet-aging (**b**) procedures of the bovine muscles *Longissimus dorsi* (LD) and *Semitendinosus (*ST).

At 48 h post-mortem (2 days), two retail cuts: ribeye steaks (*Longissimus dorsi*, LD) from the 6th to the 13th thoracic vertebrae, and the eyes of round steaks (*Semitendinosus* muscle, ST about 39 cm) from both sides of the carcasses were removed. The first retail cut (LD muscle) underwent DA and was placed into a MaturMeat^®^ (Arredo Inox S.r.l., Crotone, Italy - a European patented device and meat dry-aging method with a safe and controlled pH—n. EP 2769276B1) at the following conditions: 1 ± 1 °C, 0.5 m/s and a 78 ± 10% relative humidity (RH) for dry-aging until 60 days. Conversely, each eye of the round steaks (ST muscle) underwent WA and were cut in two sections for a total of four sub-samples sized 19.5 cm × 13 cm × 12 cm, on average. Three sub-samples (corresponding to the aging time of 15, 30 and 60 days) were weighed, vacuum packaged and placed in a cooler on a stainless-steel rack at 4 ± 1 °C. The fourth sub-sample was analyzed at 2 days. The vacuum packaging bag for the wet-aging was made of polyamide-PA and polyethylene-PE (with 20 µm of PA and 70 µm of PE in the smooth side structure; 20 µm of PA and 80 µm of PE in the embossed side structure) with an oxygen transmission rate ≤ 50 cm^3^/m^2^/24 h at 1 atm (DIN 53380—23 °C 0% RH) and a water vapor transmission rate ≤ 3.0 g^3^/m^2^/24 h at 1 atm (DIN 53122—23 °C 85% RH). The sample preparation and aging methods used are shown in Figure 1. During the aging period, the sample tests were carried out at 15, 30 and 60 days post-slaughter. At each aging time (i.e., 2, 15, 30 and 60 days), a total of eight steaks were sampled as following: two LD steaks of 4 cm thickness, two LD steaks of 3 cm thickness, two ST steaks of 4 cm thickness and two ST steaks of 3 cm thickness. Before each analysis, the muscle section of the *Longissimus dorsi* was deboned and the dried surface was trimmed. All the analyses were performed in triplicate.

### 2.2. Aging, Drip and Cooking Loss

At each sampling time (i.e., 2, 15, 30 and 60 days aging), the weight of the WA and DA meat samples were recorded before and after taking the aliquots, and the percentage of the *aging loss* was calculated at each sampling time as the difference between the initial weight and final weight divided by the initial weight and multiplied by 100.

The *drip loss* was obtained following the procedure described by Kim et al. [18]. 

For the *cooking loss*, one steak from each retail cut that was cooked for the texture profile analysis was used. The steaks were weighed before cooking at 200 °C on an open electric griddle (Farberware, Walter Idde and Co., Bronx, NY, USA). The meat was considered cooked when the core temperature of the steak reached 70 °C as verified by inserting a temperature probe (T-type, Omega Engineering, Stamford, CT, USA) into the center of each sample. The cooked samples were weighed after 24 h of refrigeration at 4 °C, and the percentage of the cooking loss was calculated using the difference between the initial and the cooked weight.

### 2.3. Physical-Chemical Analyses and Fatty Acid Profile

A proximate composition was performed only in raw meat (LD and ST) on the 2nd day after slaughtering, as no relevant compositional changes, except for the moisture, were expected within 60 days of aging. Standard procedures were used for the moisture, protein, and fat content determinations [19]. The water activity (a_w_) and pH were evaluated at each time by using Aqualab 4 TE—Decagon Devices (METER Group, Inc., Pullman, WA, USA) and a pH-meter (Crison-Micro TT 2022, Crison Instruments, Barcelona, Spain) equipped with an insertion glass electrode, respectively. The pH values were collected by placing a glass electrode at 2 cm deep into the steaks. Each measurement was performed in triplicate (*n* = 3), taking the mean value as the result. 

The intramuscular fat (IFM) was extracted in agreement with the procedures described by Hara and Radin [20], and the samples were converted into fatty acid esters according to Ambrosio et al. [21]. For the analysis of fatty acid methyl esters, gas-chromatography (DANI MASTER GC) was used with a FID detector, capillary column CP-Select CB for FAME (with a length of 100 m, internal diameter of 0.25 mm, and film thickness of 0.2 μm) and a split/splitless injector. The results are expressed as a percentage of the total fatty acids identified by using pure standard compounds (Supelco^®^ 37 Component FAME Mix, 47885U-Supelco, Sigma-Aldrich, St. Louis, MO, USA). The total saturated (SFAs), monounsaturated (MUFAs) and polyunsaturated (PUFAs) fatty acids were determined. To establish a change in the fatty acids profile during aging, the four different aging times (i.e., 2, 15, 30 and 60 days) were compared. The PUFA/SFA ratio (P/S) and the MUFA/SFA ratio (M/S) were also determined, and the atherogenic and thrombogenic indexes were calculated as follows [22]:

(1) Atherogenic Index (AI)=(4× C14:0+ C16:0)/[ΣMUFA + ΣPUFA (n −6) and (n −3)]

(2) $$\mathrm{Thrombogenic}\;\mathrm{Index}\;(\mathrm{TI})=\left(C14:0+\;C16:0+\;C18:0\right)/\left[0.5\times \;\Sigma \mathrm{MUFA}\;+0.5\times \;\mathrm{PUFA}\;\left(n\;-6\right)+\frac{n\;-3}{n\;-6\;}\right]$$

### 2.4. Lipid Oxidation (TBARs) 

A measurement of the TBARs values was performed by the extraction method as described by Xiong et al. [23] with some modifications. Specifically, after the homogenization of the meat with trichloroacetic acid and distilled water, the speed of the mixture centrifugation was set at 8000 rpm for 10 min. The TBAR_S_ value was expressed as mg/Kg of meat according to the following formula:

TBARs value (mg/Kg) = A_532_ × 7.8 (3)

### 2.5. Instrumental Color

WA and DA fresh meat samples were placed on a tray and the instrumental color was measured on the surface at three different points of the LD and ST muscles at all the aging times (i.e., 2, 15, 30 and 60 days) by using the equipment and operating conditions reported in our previously study [16].

The value of the chroma (C*) and hue angle (h°) were calculated using Equations (4) and (5), respectively:

(4) $$\mathrm{Chroma}\;(C*)=\sqrt{a^{*}{}^{2}+b^{*}{}^{2}}$$

(5) $$\mathrm{Hue}\;\mathrm{angle}\;(h{}^{\circ})=\arctan\;\frac{b^{*}}{a^{*}}\;$$

Changes in the color during post-aging were determined by the color differences coefficient (∆E) where the values of ∆L*, ∆b* and ∆a* are the differences between two aging times:

(6) $$∆E*=\;\sqrt{\left[{\left(∆L^{*}\right)}^{2}+{\left(∆a^{*}\right)}^{2}+{\left(∆b^{*}\right)}^{2}\right]}$$

### 2.6. Texture Profile Analysis (TPA) and Warner–Bratzler Shear Force (WBSF)

A texture profile analysis (TPA) and Warner–Bratzler shear force (WBSF) were performed on raw and cooked meat, at all the considered periods (i.e., 2, 15, 30 and 60 days) in agreement with Marrone et al., [16]. Particularly, at the end of the TPA test, the following parameters were analyzed: the hardness, springiness, gumminess (hardness × cohesion), chewiness (gumminess × springiness), resilience and adhesiveness. Each measurement was assessed 7–10 times and the average values were used for the statistical analysis.

### 2.7. Microbiological Analysis

To determine the logarithm concentration of spoilage microorganisms, ten grams of each WA and DA meat sample were added to 90 mL (1:10, *w*/*v*) of sterilized peptone water (PW, CM0009, OXOID, Basingstoke, UK) in a sterile stomacher bag and were homogenized at 230 rpm for three minutes in a peristaltic homogenizer (BagMixer^®^400 P, Interscience, Saint Nom, France). Subsequently, from each homogenate, ten-fold serial dilutions were prepared to isolate and enumerate the following bacteria: (i) total aerobic bacterial counts, both mesophilic and psychrophilic (TAB 30 °C and TAB 7 °C, respectively), according to ISO 4833-1:2013 and Ercolini et al. [24] on plate count agar (PCA; CM0325, Oxoid) incubated, respectively, at 30 °C for 48/72 h and at 7 °C for 10 days; (ii) total coliforms, in agreement with ISO 4831:2006 on violet-red bile lactose agar (VRBL, Oxoid, Madrid, Spain) incubated at 37 °C for 48 h; (iii) total Enterobacteriaceae, in agreement with ISO 21528-2:2017 on violet-red bile glucose agar (VRBG, Oxoid, Madrid, Spain) incubated at 37 °C for 48 h; (iv) β-glucuronidase-positive *E. coli*, in agreement with ISO 16649-2:2001 selectively isolated on tryptone bile x-glucuronide (TBX, CM0945, Oxoid, Basingstoke, Hampshire, UK) incubated at 44 °C for 24/48 h; (v) presumptive *Pseudomonas* spp., in agreement with ISO 13720:2010 on Cephalothin-sodium fusidate-cetrimide agar with a modified CFC selective supplement (CFC, CM0559B with SR0103E, Oxoid, Basingstoke, UK) incubated aerobically at 25 °C for 48 h; (vi) lactic acid bacteria (LAB), in agreement with ISO 15214:1998 on De man, Rogosa and Sharpe agar (MRS, CM0361, Oxoid, Hampshire, UK) incubated aerobically at 30 °C for 72 h; (vii) yeasts and molds on dichloran rose-Bengal chloramphenicol agar (DRBC, Oxoid, Madrid, Spain) incubated at 25 °C for 120/168 h in agreement with ISO 21527:2008; (viii) *Brochothrix* spp., in agreement with ISO 13722:2017 on streptomycin-thallous acetate-actidione (STAA) agar incubated at 22–25 °C for 48 h; and (ix) coagulase-positive staphylococci, in agreement with ISO 6888-1:1999 on Baird–Parker agar (Oxoid, Madrid, Spain) at 37 °C for 24/48 h. After incubation and counting, the data were expressed as logarithms of the number of colony-forming units (cfu/g) and the means and standard error were calculated.

To detect food pathogenic bacteria, 25 g of each WA and DA meat sample were homogenized in 225 mL (1:10 *w*/*v*) of buffer peptone water (BPW, CM0509, Oxoid, Basingstoke, UK) and incubated for 24 h at 37 °C for the detection of *Salmonella* spp., according to ISO 6579-1:2017, and in 225 mL of half Fraser broth (HF, CM1053, Oxoid, Basingstoke, Hampshire, England), incubated for 24 h at 30 °C for the detection of *Listeria monocytogenes*, according to ISO 11290-1:2017.

### 2.8. Statistical Analysis

All data were statistically analyzed using the SPSS program, version 28 (IBM Analytics, Armonk, NY, USA). ANOVA was performed by a general linear model (GLMs), including the fixed-effect of aging (for the method (DA and WA) and time (2, 15, 30 and 60 days)), the meat *states* (i.e., raw and cooked) and the interaction. The statistical significance of the comparison between the mean values was evaluated by a Tukey’s test for *p* < 0.05, *p* < 0.01 and *p* < 0.001. All the experiments were performed at least three times and the data were presented as the least square mean ± root mean square error (RMSE).

## 3. Results and Discussions

### 3.1. Carcass Traits and Meat Quality Post-Mortem

The average carcass weight was 363.6 ± 33.35 kg and the dressing percentage was 60%, in agreement with Kayar et al. [25], that considered animals of a similar live weight. Regarding the S.E.U.R.O.P. conformation and fatness score, all the carcasses were graded U2. 

Forty-eight hours post-mortem, when glycolysis is considered complete, the pH values resulted in 5.70 ± 0.05 and 5.64 ± 0.04 in the LD and ST muscles, respectively (Table 1). The differences between the muscle fibers influenced the acidification kinetics and, consequently, the pH. Indeed, the muscles are made up of slow red fibers, rich in oxidative enzymes and low in glycogen, and have a high ultimate pH compared to the other muscles formed of rapid white fibers, which have a lower ultimate pH [26,27]. For this reason, immediately after slaughter, an evident difference between the forequarter (FR) and hindquarter (HD) pH values was detected, with significantly higher pH values in the FRs (Table 1). These results are in agreement with those of Stella et al. [28] who observed the same trend for the whole aging period. Glycolytic changes in the muscles can be followed using pH measurements, and a decrease of the meat pH from 7.0 to 5.5, for example, is essential for a reduction in bacterial growth [29] and for the stability of meat. For the proximate composition (Table 1), no significant differences between the ST and LD muscles were detected except for the intramuscular fat (IMF) content that was higher (*p* < 0.05) in the LD, compared to the ST muscle. 

### 3.2. Effect of Post Aging Methods on Meat pH, Water Activity (a_w_) and Water-Holding Capacity (WHC) 

The effects of the post-aging method on the physical characteristics of meat are reported in Table 2. Some authors [13,30] have shown that during 60 days of dry-aging, the pH value increased as a result of the production of nitrogenous compounds of proteolysis. In our study, the pH value did not significantly change during the DA, according to a previous study [16]. The WA meat showed a lower final pH compared to the DA meat (*p* < 0.05), and this might be due to the vacuum bag used in the WA that provided a favorable environment which allowed microbial growth. The a_w_ tended to decrease during the 60 days of the DA period, but the difference was not significant (*p* > 0.05). This finding was in agreement with Cho et al. [13]. 

The water holding capacity (WHC) was assessed by measuring a series of assays including the aging loss, drip loss and cooking loss of the meat samples (Table 2). Indeed, a WHC determination is useful to estimate the important quality traits of raw meat such as juiciness. Dashdorj et al. [17] reported that beef dry-aging generally results in a 30–40% overall aging loss, mainly due to moisture evaporation during the aging period, resulting in a higher economic loss. In our study, the overall aging loss of the DA after 60 days was lower than 20%. In contrast, the WA showed a lower aging loss (around 5%) at the end of aging (Table 2). At 30 days of DA, the aging loss remained constant and then increased afterwards. This result could be explained by the protection offered by the vertebras (6th–13th) and by the coverage of subcutaneous fat from the excessive evaporation of surface moisture during the 60 days of DA. As regards the aging loss of the WA meat for 60 days, the results were in agreement with those of Kim et al. [30].

The drip loss, namely, the amount of free water exudated during storage, is correlated to several aspects such as the water permeability of the cell membrane and the pH-induced protein denaturation [31]. In particular, a greater protein denaturation could justify the highest drip loss after 48 h post-mortem. Indeed here, the aging time did not significantly change the drip loss of the DA meat, and only the WA meat was decreased from 2.70 to 1.40 after 60 days (Table 2). During the aging process, complex reactions that occur on the myofibrillar proteins (proteolysis) [32] and changes in collagen [33] can modify the muscle structure. These structural changes significantly affect the ion–protein interactions, increasing the capillary space accessible to water [34]. The decrease in microcapillary space diameters reduces the WHC of proteins and, hence, most of the water is lost during the dry-aging of meat. Indeed, as shown by our results, the water loss during the meat aging had the inverse trend of the drip loss.

Cooking loss is the amount of water lost from meat during cooking, and it is essentially due to protein denaturation [35]. It is directly associated with the texture properties and eating quality [36] because moisture loss during cooking can lead to a loss of flavor compounds, thus, negatively affecting chewing. There were no significant differences in the cooking loss over time here, as also reported by Utama et al. [37]; however, the WA meat showed a significantly (*p* < 0.05) higher cooking loss than the DA meat, which was more accentuated (*p* < 0.01) at 60 days of aging (Table 2). These results were in agreement with those of Ozawa et al. [38] and suggest that an elevated cooking loss was significantly lower in the highest marbling score (i.e., intramuscular fat content) samples. In contrast, Kim et al. [39] showed that the intramuscular fat content did not influence cooking loss. In our study, the significant differences in the intramuscular fat content (Table 1) could be the cause of the differences in the cooking loss. Some authors [40,41] have reported, however, that dry-aging reduces the cooking loss regardless of the meat cut. This finding shows that DA beef has a higher water-holding capacity than WA beef during cooking. 

### 3.3. Effect of Post-Aging Methods on Instrumental Color and Lipid Oxidation (TBARs)

**Figure 2:**
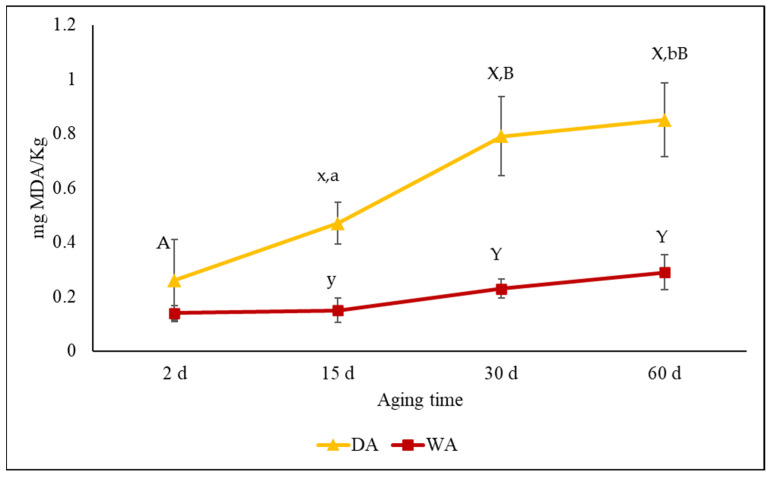
Changes in thiobarbituric acid reactive substances value (mg of malondialdehyde/kg) of Charolais meat cuts (*Longissimus dorsi* (LD) and *Semitendinosus* (ST)) aged by different methods (dry-aging (DA) and wet-aging (WA)) for 60 days.

The color evaluation is important to understand the beef quality and its value [42], but during the aging process, several changes that occur in beef muscles are responsible for color changes [43]. In dry-aged meat, a prolonged exposure to oxygen not only promotes metmyoglobin formation, but also intramuscular lipid oxidation [43,44]. The high content of intramuscular fat in the DA anatomical cut and the exposition to oxygen, affected the TBARs values that were significantly (*p* < 0.01) higher in the DA than in the WA meat after 15 days of aging, as also reported by Kim et al. [30]. It is known that TBARs values higher than 2 mg MDA/kg of meat can negatively affect the eating quality due to the lipids oxidation, off-flavor and taste [45]. Although the TBARs values increased significantly (*p* < 0.01) during the DA (Figure 2), they were lower than 0.9 mg of MDA/kg of a sample after 60 days of aging. Even though a previous study [30] reported TBARs values higher than 2 mg MDA/kg of dry-aged sirloin beef after 45 days of aging, in our study, the overall TBARs values of the DA meat could be explained by the protection provided by the vertebral bone and fat coverage in the loins. Instead, the WA method ensured a greater oxidative stability resulting from the low values of the TBARs compared to DA (Figure 2). This aspect could be associated with the low oxygen transmission rate of the vacuum packaging bag which had a positive effect by preserving the lipids from the oxidative processes [44]. It is known that the accumulated products of lipid oxidation promote myoglobin oxidation and, consequently, changes in the meat color [46], playing an important role in the discoloration of steaks. 

The aging period, as well as the aging method, had no significant effect on color changes, except for the lightness (Table 3). Generally, the meat color was affected by the muscle type but no significant differences were observed between the DA and WA cuts at 2 days, confirming the results shown by Modzelewska-Kapituła et al. [47]. The intramuscular fat content may also affect the color parameters of different muscles at various aging times. According to Silva et al. [48], a lower intramuscular fat content was associated with lower L* and b* values in wet-aged beef steaks, but our results do not confirm the latter finding. During the DA, the meat color changed in different ways, namely, in the first 30 days the a*, b* chroma (C*) and hue angle (h°) tended to decrease while the lightness (L*) tended to increase (without reaching the significance). Extending the period by a further 30 d, the WA meat color did not change while the DA meat became brighter (Table 3). In the DA cuts, these results could be explained by vertebral and subcutaneous fat coverings that protected the muscles from denaturation, thus ensuring a stable color. In the same way, the low oxygen transmission rate of the vacuum packaging bag in the WA cuts of meat did not lead to lipid oxidation and the color did not change; however, the vacuum packaging bag determined a temporary browning of the meat that regained its color when the vacuum bag was opened. Marrone et al. [16], studying the effects of the dry-aging process on two anatomical cuts (i.e., LD and ST), reported that the ST muscle after a prolonged DA process of > 30 days became darker and the color uniformity became less desirable. Our study allowed a small size muscle without fat coverage, such as the ST, to be aged for up to 60 days while obtaining desirable characteristics; however this was possible with changing the aging method and using the wet-aging process.

**Figure 3:**
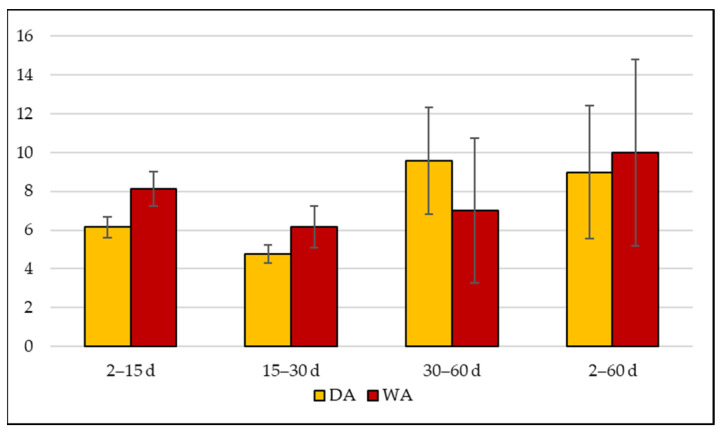
Variation of color (∆E) during dry (DA) and wet (WA) aging in two Charolais meat cuts (*Longissimus dorsi* (LD) and *Semitendinosus* (ST)) aging assessed between successive measurements (2 days and 15 days; 15 days and 30 days; 30 days and 60 days; 2 days and 60 days).

Changes in the meat color during aging were also evaluated using the ΔE coefficient. In the present study, the ΔE coefficient was expressed as the difference between successive measurements (i.e., 2 days and 15 days; 15 days and 30 days; 30 days and 60 days), and was always > 3 (Figure 3). According to Francis et al. [49], when the ΔE  >  3, the color differences are obvious for human eyes. Our results then indicate that the color differences measured by the colorimeter were perceptible to the human eye. As shown in Figure 3, the WA meat was characterized by an accentuated change in color in the first 15 days of aging, which continued to be perceptible as the aging time increased. In contrast, in the DA meat, the perception of the color change was very high between 30 and 60 days.

### 3.4. Effect of Post-Aging Methods on Warner–Bratzler Shear Force (WBSF) and Texture Profile Analysis (TPA)

An evaluation of the texture traits of raw and cooked meat is important to optimize the aging process and achieve desirable textural characteristics. Several factors influence the tenderness of the meat, including the aging time and the type of muscle. The different biochemical characteristics of muscles could influence the response to the aging process [12]. At 48 h post-mortem, the WA meat showed significantly higher values than the DA meat (*p* < 0.05) for almost all texture parameters (Table 4). In agreement with Vaskoska et al. [50], the DA cuts showed lower values of hardness, gumminess and chewiness compared to the WA cuts. The differences between the two aging methods were present for the TPA measurements up to 30 days in the raw meat, except for the chewiness values that were always different; however, for these values, in the cooked meat, the aging method showed no effects starting from 15 days (with no significant differences between the WA and DA meats). The significant difference in the amount of intramuscular fat between the two muscles (Table 1) might have been an important factor that affected these parameters, in accordance with Lin Xu et al. [12]. Although the *Semitendinosus* muscle is usually recognized as being a tough muscle [6], our results suggest that 15 days of WA can improve the hardness and chewiness of this muscle. No difference in the resilience was detected among the aging methods or the aging times with each aging method. The hardness in the aged meat significantly (*p* < 0.01) decreased after 30 days of DA, while it decreased after 15 days in the WA. The adhesivity and gumminess in the aged meat performed similar to the hardness factor. Overall, extending the aging time appeared to standardize the physical-chemical characteristics of the two cuts considered, in agreement with Wyrwisz et al. [51]. In particular, in our study, meat samples after 15 days of aging were more tender than for 2 days, regardless of the aging method. This result implies that the aging time and raw material quality grade are important factors that could improve meat tenderness regardless of the aging method, in agreement with Kim et al. [30]. 

The WBFS test, in contrast to the TPA results, showed that already after 15 days of aging there were no differences between the aged cuts and in particular, at 30 days, both reached the lowest WBSF value (Table 4). Kim et al. [30] showed a gradual decrease in the WBSF value of wet-aged meat during 60 days of aging (from 6.36 to 3.10 kg) compared to dry-aged and packed-dry-aged meat which showed a less regular decrease. 

Our results showed that the cooking process increased all the texture traits typical of meat toughness (Table S1). After cooking the meat, a significant increase in the WBSF in the LD and in the hardness in both muscles was registered (Table 4). In particular, the hardness value in the LD, aged by DA, was increased by 200% at 15 and 30 days, while in the ST, aged by WA, the amount improved to just over 60%, except at 30 days when the increase was more contained (28%). This could have been due to the different content of the connective tissue and collagen of the two muscles. According to Warner et al. [52], the TPA test on cooked meat (at 70 °C at its core), was sensitive to the properties of the intramuscular connective tissue. Indeed, the TPA test reflected the connective tissue contribution better than the shear test during a cooked meat tenderness measurement [52]. On the other hand, muscles aged for 30 and 60 days showed the same response to the cooking, by highlighting the overlapping results for rheological traits in DA and WA cuts post-cooking. This highlights how extending the aging beyond 30 days could represent a disadvantage rather than an advantage, regardless of the type of muscle or aging method.

### 3.5. Effect of Post-Aging Methods on the Fatty Acid Profile

The changes in the fatty acid composition during 60 days of DA and WA are shown in Table 5. A study by [37] was previously carried out to investigate changes in the fatty acids profile as a result of the aging time and method. In our study, no significant interaction was found, except for C14:0, that resulted in being higher at 2 and 60 days of aging and lower at 15 days in DA meat, compared to WA meat (*p* < 0.05). The fatty acid profile of the WA meat showed less changes compared to the DA meat during aging. The stearic acid (C18:0) increased in the DA meat and in the same way increased the content of C18:1 trans at the end of the DA. In contrast, oleic acid (C18:1n9 cis) and palmitoleic acid (C16:1) were greater in the meat before aging and decreased during the DA. A declining proportion of oleic acid after dry-aging was also observed in a previous study [37]. It is known that *cis* fatty acids are more susceptible to oxidation than *trans* fatty acids, and this aspect allows us to hypothesize that the prolonged exposure to oxygen during the dry-aging process may have degraded the *cis* fatty acids more rapidly. For this reason, the extension of the DA period could be responsible for the declining proportion of monounsaturated fatty acid (MUFA) and polyunsaturated fatty acids (PUFA) and the increase in saturated fatty acids (SFA), compared to WA meat. The aging method seems to have greatly influenced the fatty acid profiles suggesting that the principal changes were due to differences in the oxidative stability. This aspect could be explained by the low oxygen transmission rate of the vacuum packaging bag which had a positive effect by preserving the fatty acids from oxidative processes [44].

### 3.6. Effect of Post-Aging Methods on the Microbiological Profile

Meat is a favorable substrate for different bacterial strains. The stages of slaughtering, cutting, processing, transport and storage may influence the growth of several bacterial populations and, consequently, affect meat conservation and maturation [53]. The dry-aging process is characterized by the presence of beneficial molds, whose development, after three weeks from the beginning of the aging process, and subsequent penetration into the meat, involves the release of proteases that destroy muscles and connective tissues [17]. In the present study (Table 6), an important presence of yeasts and molds was detected already at the first sampling time, respectively, of 2.8 and 1.6 Log (CFU/g); this data could presumably be related to a cross-contamination from the presence of these organisms on the work tables. It is worth noting there was an increase in the mold levels in the DA samples at 15 days of aging, followed by a downward trend throughout the aging period. This first result could be explained by considering a possible contamination of the maturation room in which the DA samples were housed; this condition could have influenced the mold concentration 15 days after slaughter.

Although it is well known that the packaging in the wet-aging system acts as a physical barrier to external micro-organisms such as yeasts and molds [54], no significant logarithmic differences were found between the DA and WA meat samples. This finding is probably due to the presence of these micro-organisms on the inner surface of the vacuum bag or in the environment where the sample preparation was carried out. Nevertheless, the yeast levels of both the sample types (i.e., the WA and DA) were found to be significantly affected by the aging period, showing similar trends in both the DA and WA samples (*p* < 0.001). 

Previous studies have reported a greater presence of lactic acid bacteria (LAB 30 °C) in WA meat, in correlation to a high percentage of superficial moisture and a low presence of oxygen [54,55]. Obviously, according to this assumption, the concentration of bacteria, as well as of LAB, should be lower in DA than in WA meat; however, this hypothesis does not consider the properties of the aging chambers used in the present study. Indeed, as reported above in the material and methods section (Section 2.1), the MaturMeat^®^ (Arredo Inox S.r.l., Crotone, Italy) allows the selection of a specific climatic recipe, including the RH which was set at 78%. This percentage of RH allows for the controlling and slowing down of the process of dehydration of the external surface; therefore, the chemical analyses showed no significant differences in the a_w_ (Table 2) between the DA and WA samples. Furthermore, the vacuum bags’ permeability to oxygen and water plays a key role in the comprehension of bacteria growth trends in WA samples. As reported above, the vacuum bags used in this study were expected to be a good oxygen barrier, having a value of the oxygen transmission rate (OTR) of about 50 cm^3^/m^2^ day 1 atm. In this regard, it is worth considering that this value refers to measurements carried out at 0% RH. According to several authors [56,57], the barrier behavior of polymers, such as polyamide (PA) and polyethylene (PE), is influenced by several factors, including the RH and the period of food storage. In particular, it is well known that PA/PE polymeric films may lose their oxygen-barrier properties when the environmental RH increases [56,57]. In our case study, the RH of the storage chambers, in which the WA samples were housed, was approximately 65–70%, higher than that used to measure the OTR of these vacuum bags. This consideration could justify the non-significant differences in the concentration of *Pseudomonas* spp. and Lactic acid bacteria between the WA and DA samples. If the packaging of the WA samples had totally preserved its barrier role to oxygen, a decrease in these aerobic microorganisms would have occurred. Nevertheless, an interesting result was observed as concerning the concentration of LAB in the WA samples on the 30th and 60th days after aging, when a decrease in these bacteria wasdescribed when compared to the value recorded on the 15th day of aging. These findings would likely suggest that, even though the OTR of the vacuum bag during the experiment was presumably higher than that provided in the product data sheet, there was a partial preservation of the oxygen barrier capability of the vacuum bag. Overall, it is possible to assume that the vacuum bag used to package the WA samples hindered the availability of oxygen to bacteria; however the storage conditions affected the OTR value and, therefore, almost nullified the differences between the WA and DA samples.

According to the FAO/WHO, the total aerobic bacteria (TAB 30 °C) count could be used as a parameter for refrigerated meat to evaluate the hygienic conditions in which it was produced [58]. However, a common limit of TAB has not been established for aged meat, perhaps due to the multitude of factors that could differently influence the degenerative processes in meat. In fact, it is known that especially for packaged meat, the alterations are generally related more to the nature of the microbial flora than to the number of bacteria present [58]. Indeed, in this study, although a high TAB 30 °C concentration was detected in both the DA and WA samples, it is possible to hypothesize a flaw in the application of good hygiene practices (GHP) when the levels of Enterobacteriaceae and Coliforms are also taken into account. Moreover, the presence of *E.coli* in the DA and WA samples 48 h after slaughter (*p* < 0.01) supported the previous hypothesis and highlights the importance of the workers’ formation and the application of GHP, which is reflected in the hygiene and safety of the products. 

Pathogenic bacteria such as *Salmonella* spp., *Listeria monocytogenes* and *Staphylococcus aureus* were not isolated in either of the WA or DA meat during all the sampling times.

## 4. Conclusions

Presently, wet-aging and dry-aging are the two main forms of aging methods used by local processors and food retailers to satisfy consumer tastes; however, the two methods are not suitable for all the anatomical cuts. Our study has shown that choosing the aging method considering the chemical characteristics of the muscle is a strategy to obtain optimal results. Overall, an aging period of more than 15 days and the interactive effect with a suitable aging method would seem to standardize the physical characteristics of the two cuts (i.e., *Semitendinous* and *Longissimus dorsi*), while not negatively influencing the quality traits, and also containing losses. In this regard, both wet-aging and dry-aging improved the quality of different beef cuts, suggesting the best method–time aging combination for the beef industry and consumers’ preferences.

## Figures and Tables

**Table 1:** Carcass traits and proximate composition of *Longissimus dorsi* (LD) and *Semitendinosus* (ST) Charolais muscles after 48 h of slaughter (mean value ± S.D; *n* = 3/muscle).

Items	Carcass Traits and Meat Quality	
Immediately post-slaughter	FR	HD
pH of quarters	6.86 ± 0.22 ^A^	6.58 ± 0.19 ^B^
After 48 h of slaughter	LD	ST
Moisture, %	69.32 ± 1.65	75.54 ± 1.21
IFM, %	6.90 ± 1.02 ^a^	2.45 ± 0.43 ^b^
Protein, %	14.88 ± 2.66	20.64 ± 1.04
pH^48^	5.70 ± 0.05	5.64 ± 0.04

FR: forequarters, HD: hindquarters and IFM: intramuscular fat. Different superscript letters indicate a significant difference at *p* < 0.05 (lowercase letters) and *p* < 0.01 (capital letters).

**Table 2:** Effect of time and method of aging on some physical features of Charolais meat cuts (*Longissimus dorsi* (LD) and *Semitendinosus* (ST)) aged by different methods (dry-aging (DA) and wet-aging (WA)) for 60 days.

		Aging Time, Days					Effect		
Items	Method	2	15	30	60	RMSE	T	M	T × M
pH	DA	5.70	5.64	5.66 ^x^	5.72 ^x^				
	WA	5.64	5.65	5.82 ^y,A^	5.54 ^y,B^	0.05	NS	NS	*
a_w_	DA	0.982	0.973 ^x^	0.980	0.976				
	WA	0.981	0.982 ^y^	0.979	0.974	0.00	NS	*	NS
Aging loss, %	DA	-	4.47 ^A^	3.20 ^A^	9.37 ^X,B^	0.76			
	WA	-	2.95 ^A^	3.73 ^B^	4.50 ^Y^		**	NS	***
Drip loss, %	DA	2.47	1.77	1.46	1.15	0.33			
	WA	2.70 ^a^	1.78	1.40	1.10 ^b^		**	NS	NS
Cooking loss, %	DA	15.85 ^x^	12.99 ^x^	19.22	12.62 ^X^	2.89			
	WA	24.93 ^y^	24.15 ^y^	27.12	24.37 ^Y^		NS	***	NS

T: aging time and M: aging method. Statistical analysis was performed comparing the experimental groups (DA and WA) at each aging time (2, 15, 30 and 60 days) and within each experimental group along the aging period. The a,b mean values in the same row (aging time) with different letters differ significantly for *p* < 0.05 (lowercase) or *p* < 0.01 (capital). The x,y mean values in the same column (different samples on the same aging time) differ significantly for *p* < 0.05 (lowercase) or *p* < 0.01 (capital). NS: not significant; * = *p* < 0.05; ** = *p* < 0.01; *** = *p* < 0.001.

**Table 3:** Changes in the meat color attributed as a function of the aging method and time in Charolais meat cuts (*Longissimus dorsi* (LD) and *Semitendinosus* (ST)) aged by different methods (dry-aging (DA) and wet-aging (WA)) for 60 days.

		Aging Time, Days					Effect		
Items	Method	2	15	30	60	RMSE	T	M	T × M
Lightness, L*	DA	40.16	43.44	39.18	47.01	2.63	*	NS	NS
	WA	35.22	42.32	39.02	44.80				
Redness, a*	DA	16.21	14.75	15.18	16.71	1.46	NS	NS	NS
	WA	14.86	16.71	14.45	15.94				
Yellowness, b*	DA	14.99	14.27	13.25	14.53	0.82	NS	NS	NS
	WA	13.64	13.94	11.45	14.48				
Chroma, C*	DA	22.09	20.59	20.19	22.22	1.37	NS	NS	NS
	WA	20.18	21.84	18.46	21.56				
Hue angle. h°	DA	42.62	44.33	41.52	40.95	2.62	NS	NS	NS
	WA	42.50	40.54	38.44	42.53				

T: aging time and M: aging method. Statistical analysis was performed comparing the experimental groups (DA and WA) at each aging time (2, 15, 30 and 60 days) and within each experimental group along the aging period. NS: not significant; * = *p* < 0.05.

**Table 4:** Changes in texture profile analysis (TPA) and Warner–Bratzler shear force (WBSF) as a function of the aging method and time in Charolais meat cuts (*Longissimus dorsi* (LD) and *Semitendinosus* (ST)) aged by different methods (dry-aging (DA) and wet-aging (WA)) for 60 days.

		Aging Time, Days								
Items	Method	2		15		30		60		RMSE
		Raw	Cooked	Raw	Cooked	Raw	Cooked	Raw	Cooked	
WBSF, Kg	DA	2.00	2.60	3.23	2.85	1.94	2.40	2.36	2.51	
	WA	4.35 ^a^	2.98	3.65 ^ab^	3.06	1.42 ^b^	1.38	3.06 ^ab^	3.23	0.65
		*								
Hardness, N	DA	23.05 ^X^	51.06 ^Y^	18.55 ^X^	56.70 ^Y^	16.85 ^X^	55.43 ^Y^	25.03 ^X^	58.99 ^Y^	2.02
	WA	47.59 ^A^	77.46	38.54 ^X^	63.12 ^Y^	56.69 ^B^	72.82	43.71 ^x^	72.58 ^y^	
			**	*		**				
Gumminess, N	DA	7.65 ^X^	22.43 ^Y^	5.89 ^X^	23.39 ^Y^	5.37 ^X^	23.49 ^Y^	7.31 ^X^	23.07 ^Y^	0.81
	WA	16.76 ^X^	38.42 ^Y,a^	13.85 ^X,A^	28.22 ^Y,b^	23.38 ^aB^	30.62	15.48 ^b^	31.05	
		*	*	**	**	**				
Chewiness, N × mm	DA	5.65 ^X^	15.50 ^Y^	4.26 ^X^	17.81 ^Y^	3.77 ^X^	17.91 ^Y^	5.30 ^X^	18.83 ^Y^	
	WA	13.87 ^X,A^	31.58 ^Y^	12.27 ^X^	23.24 ^Y^	18.02 ^B^	23.01	12.43 ^X,A^	26.08 ^Y^	0.69
		**	**	**		**		**		
Springiness, mm	DA	0.73	0.69 ^aA^	0.73	0.76 ^b^	0.72	0.76 ^B^	0.72	0.82 ^b^	
	WA	0.82	0.83	0.88	0.83	0.83	0.79	0.82	0.84	0.01
		*	**	**	*			*		
Resilience	DA	1.78	0.09	2.49	0.11	1.44	0.09	2.13	0.11	
	WA	0.22	0.18 ^A^	0.27	0.18 ^a^	0.25	0.29 ^bB^	0.23	0.21	0.24
			**		*		**		*	
Adhesiveness	DA	−3.48 ^a^	−6.63 ^a^	−2.68	−12.15	−3.78	−14.28	−6.44 ^x,b^	−30.60 ^y,b^	1.21
	WA	−5.03	−17.69	−6.81 ^x^	−11.46 ^y^	−5.68	−12.44	−6.35	−23.18	

Statistical analysis was performed comparing the aging methods and meat *states* (DA and WA; raw and cooked) at each aging time (2, 15, 30 and 60 days) and within each experimental group along the aging period. The a,b mean values in the same row (aging time) with different letters differ significantly for *p* < 0.05 (lowercase) or *p* < 0.01 (capital). The x,y mean values in the same column (with different samples for the same aging time) differ significantly for *p* < 0.05 (lowercase) or *p* < 0.01 (capital). Different symbols within a column indicate that the means differ significantly for *p* < 0.05 (*) or *p* < 0.01 (**).

**Table 5:** Changes in the fatty acid composition of meat as a function of the aging method and time in Charolais meat cuts (*Longissimus dorsi* (LD) and *Semitendinosus* (ST)) aged by different methods (dry-aging (DA) and wet-aging (WA)) for 60 days.

		Aging Time, Days					Effect		
Items	Method	2	15	30	60	RMSE	T	M	T × M
C14:0	DA	2.32 ^x^	1.72 ^X^	1.85	2.12 ^x^	0.15	*	NS	***
	WA	1.72A ^y^	2.98 ^Y,B^	2.09 ^A^	1.65 ^y,A^				
C16:0	DA	21.07	20.25	24.76 ^x^	23.32	1.48	NS	NS	NS
	WA	19.06	19.45	19.66 ^y^	23.94				
C16:1	DA	3.06	2.31 ^X^	2.79	2.83	0.42	NS	*	*
	WA	2.83	3.81 ^Y^	3.56	2.98				
C17:0	DA	1.41	1.42	1.51	1.38	0.16	NS	NS	NS
	WA	1.31	1.59	1.45	1.65				
C18:0	DA	16.71	20.92 ^x^	18.93	18.43	1.88	NS	*	NS
	WA	15.76	15.00 ^y^	16.10	16.34				
C18:1n9 *trans*	DA	2.55	3.08	2.15	3.33	0.45	NS	NS	NS
	WA	2.83 ^a^	4.20	2.97	2.14 ^b^				
C18:1n9 *cis*	DA	46.11	44.13	42.43	40.32 ^X^	1.73	NS	**	NS
	WA	48.47	46.44	46.22	48.19 ^Y^				
C18:2n6 *cis*	DA	3.25	3.27	2.84	2.60	0.39	NS	*	NS
	WA	3.58	3.67	3.89	3.65				
C18:3n3	DA	0.40 ^a^	0.32 ^x^	0.25 ^b^	0.29	0.03	***	NS	NS
	WA	0.40 ^Aa^	0.20 ^y,B^	0.26 ^b^	0.28				
CLA	DA	0.30	0.31	0.30	0.26	0.04	NS	NS	NS
	WA	0.41	0.28	0.33	0.29				
C20:4n6	DA	0.40	0.24	0.16	0.28	0.06	NS	**	NS
	WA	0.40	0.55	0.43	0.38				
∑SFA	DA	42.48	45.22 ^x^	47.85 ^X^	45.25	1.76	NS	***	NS
	WA	39.52	39.03 ^y^	39.31 ^Y^	43.58				
∑MUFA	DA	52.99	50.56	48.52	46.48 ^x^	1.69	NS	**	NS
	WA	55.16	54.45	52.75	53.32 ^y^				
∑PUFA	DA	4.44	4.23	3.63 ^x^	3.43	0.42	NS	NS	NS
	WA	4.88	4.70	4.91 ^y^	4.60				
n6	DA	3.65	3.52	2.99 ^x^	2.88	0.44	NS	*	NS
	WA	3.98	4.22	4.32 ^y^	4.03				
n3	DA	0.49	0.32	0.25	0.45	0.09	*	NS	NS
	WA	0.66	0.36	0.42	0.44				
AI	DA	0.53	0.50	0.62	0.65	0.04	NS	*	NS
	WA	0.43	0.53	0.49	0.54				
TI	DA	1.41	1.58	1.77 ^x^	1.79	0.12	NS	**	NS
	WA	1.23	1.27	1.32 ^y^	1.48				

T: aging time and M: aging method. Statistical analysis was performed comparing experimental groups (DA and WA) at each aging time (2, 15, 30 and 60 days) and within each experimental group along the aging period. The a,b mean values in the same row (post-aging time) with different letters differ significantly for *p* < 0.05 (lowercase) or *p* < 0.01 (capital). The x,y mean values in the same column (with different samples for the same aging time) differ significantly for *p* < 0.05 (lowercase) or *p* < 0.01 (capital). NS: not significant; (*) *p* < 0.05; (**) *p* < 0.01; (***) *p* < 0.001.

**Table 6:** Effects of different aging methods and aging times on the microbial population (log colony-forming unit (cfu)/g) in Charolais meat cuts (*Longissimus dorsi* (LD) and *Semitendinosus* (ST)) aged by different methods (dry-aging (DA) and wet-aging (WA)) for 60 days.

		Aging Time, Days					Effect		
Items	Method	2	15	30	60	RMSE	T	M	T × M
TAB 30 °C	DA	4.10	5.45	5.31	5.38	0.23	NS	NS	NS
	WA	3.74	4.15	5.43	5.11				
TAB 7 °C	DA	2.27	4.33	3.29	3.58	0.31	NS	NS	NS
	WA	3.48	4.78	3.88	3.71				
Total coliforms	DA	3.08	3.52	1.75	3.93	0.26	NS	NS	NS
	WA	3.25	2.64	3.12	3.45				
Enterobacteriaceae	DA	1.78	2.28	1.73	1.92	0.26	NS	NS	NS
	WA	1.49	1.83	2.83	1.21				
β-glucuronidase-positive *E. coli*	DA	1.33	ni	ni	ni	0.12	**	NS	NS
	WA	0.95	ni	ni	ni				
*Pseudomonas* spp.	DA	2.06	3.89	2.65	3.18	0.27	NS	NS	NS
	WA	3.08	3.17	3.56	2.66				
LAB 30 °C	DA	2.41	3.00	2.94	3.23	0.14	NS	NS	NS
	WA	3.03	4.23	3.53	3.41				
Yeast	DA	1.65	2.76	ni	1.81	0.18	***	NS	NS
	WA	Ni	1.65	ni	2.47				
Mold	DA	2.81	3.59	2.81	2.84	0.26	NS	NS	NS
	WA	2.66	4.12	3.60	3.77				

T: aging time and M: aging method. Statistical analysis was performed comparing the experimental groups (DA and WA) at each aging time (2, 15, 30 and 60 days) and within each experimental group along the aging period. ni: not isolated; NS: not significant; (**) *p* < 0.01; (***) *p* < 0.001.

## Data Availability

The data presented in this study are available on request from the corresponding authors.

## Supplementary Materials

The following supporting information can be downloaded at: https://www.mdpi.com/article/10.3390/foods12030531/s1, Table S1. Effects of variables (T = aging time, M = aging method and S = states of meat (raw or cooked)) on the changes in the texture profile analysis (TPA) and Warner–Bratzler shear force (WBSF).
