# Supplementary material for: Effects of the Aging Period and Method on the Physicochemical, Microbiological and Rheological Characteristics of Two Cuts of Charolais Beef
Source: Foods. 2023 Jan 25;12(3):531. doi: 10.3390/foods12030531 (PMC9914319; doi:10.3390/foods12030531)
Supplement: Supplementary file 1 [file foods-12-00531-s001.zip › foods-2163770-supplementary.pdf]

**Table S1.** Effects of variables (T = aging time, M = aging method and S = states of meat (raw or cooked)) on the changes in texture profile analysis (TPA) and Warner-Bratzler shear force (WBSF).

| | Effect | | | | | |
|---|---|---|---|---|---|---|
| | **T** | **M** | **S** | **T×M** | **T×S** | **T×M×S** |
| WBSF, Kg | ** | NS | *** | NS | NS | NS |
| Hardness, N | NS | *** | *** | NS | NS | NS |
| Gumminess, N | NS | *** | *** | NS | NS | NS |
| Chewiness, N x mm | NS | *** | *** | NS | NS | NS |
| Springiness, mm | NS | *** | NS | NS | NS | NS |
| Resilience | NS | NS | NS | NS | NS | NS |
| Adhesiveness | NS | NS | *** | NS | NS | NS |

NS = not significant; ** = $p < 0.01$; *** = $p < 0.001$.
